# Collagen type 1 alpha 1 chain is a novel predictive biomarker of poor progression-free survival and chemoresistance in metastatic lung cancer

**DOI:** 10.7150/jca.59723

**Published:** 2021-07-25

**Authors:** Lingjie Hou, Tie Lin, Yicun Wang, Bao Liu, Meng Wang

**Affiliations:** 1 Department of Respiratory Medical Oncology, Harbin Medical University Cancer Hospital, Heilongjiang, Harbin 150081, China; 2 Department of Surgery, The First Affiliated Hospital of Harbin Medical University, Heilongjiang, Harbin 150001, China.; 3 Jilin Provincial Key Laboratory on Molecular and Chemical Genetic, The Second Hospital of Jilin University, Jilin 130041, Changchun, China

**Keywords:** Extracellular matrix protein, *COL1A1*, Chemoresistance, Metastatic, Lung cancer

## Abstract

**Background:** Collagen type 1 alpha 1 chain (*COL1A1*) is an extracellular matrix protein comprising two alpha 1 chains and one alpha 2 chain. Our previous study identified that *COL1A1* is the key gene during the development and progression of lung adenocarcinoma by multi-omics analysis. However, the clinical significance of *COL1A1* expression in lung cancer samples remains largely unknown. Here, we aimed to evaluate the level of *COL1A1* in lung cancer samples and correlate its level with the clinical outcome.

**Methods:***COL1A1* gene expression in lung cancer samples was analyzed using the Oncomine database (www.oncomine.org). A total of 308 lung cancer samples (208 formalin-fixed paraffin-embedded tissues and 100 blood samples) were assessed for protein expression of *COL1A1*. Immunohistochemistry staining and enzyme-linked immunosorbent assay were used to detect *COL1A1* expression in tissues and serum, respectively.

**Results:** We identified an elevation of *COL1A1* in mRNA level and gene amplification in lung cancer tissues compared with normal lung tissues. High *COL1A1* expression was observed in lung cancer tissues and serum (*P* < 0.05), it was significantly correlated with the peripheral type tumor, the larger diameter of the tumor, the occurrence of lymph node metastases and distant metastases, a higher TNM stage, and smoking (*P* < 0.05). High *COL1A1* expression was associated with poor progression-free survival (PFS) and chemoresistance in lung cancer patients (*P* < 0.05). Multivariable Cox-regression analysis showed that *COL1A1* expression was an independent prognostic factor (*P* < 0.05). Furthermore, the area under the receiver operating characteristic (AUC) curve was 0.909 for the combined *COL1A1* and carcinoembryonic antigen (CEA) measurement.

**Conclusion:** Our findings revealed that *COL1A1* could be used as a novel diagnostic, prognostic, and chemoresistance biomarker of human lung cancer, and these results provide a potential therapeutic strategy for lung cancer patients.

## Introduction

Lung cancer is one of the most common malignant tumors and the leading cause of cancer deaths worldwide 1. Genetic factors and environmental exposure impact the development and progression of this complex disease. Smoking and various susceptibility genes associated with lung cancer have been considered a possible mechanism for the development of lung cancer 2. However, the biological risk factors of lung cancer aggressiveness and chemoresistance remain elusive. In our previous research, we identified collagen type 1 alpha 1 chain (*COL1A1*) as the key gene during the development and progression of lung adenocarcinoma (LUAD) by multi-omics analysis 3.

*COL1A1* is a component of the extracellular matrix (ECM), and its gene is located on chromosome 17 (*17q21.23*) and contains the myopia 5 (*MYP517q21-22*) locus 4, 5. This gene encodes the pro-alpha 1 chain of type I collagen, which has a triple helix comprising two alpha 1 chains and one alpha 2 chain 6. Specifically, *COL1A1* mutations can lead to collagen-related diseases, including autosomal dominant osteogenesis imperfecta (OI) and the rare arthrochalasia subtype of Ehlers-Danlos syndrome (EDS) 7. Recently, it has been shown that a high level of *COL1A1* is indicative of a more aggressive cellular behavior and poorer prognosis in patients with breast cancer, especially in those with ER+ breast cancer 8. Furthermore, *COL1A1* expression was correlated with the key genes of the WNT/PCP signaling pathway, which may promote colorectal cancer cell migration 9. In mouse models, *COL1A1* was frequently upregulated in gastric cancer tissues; it increased cell proliferation, colony-forming efficiency, migration ability, and invasion ability, while it promoted the development of grafted tumors in mice 10. However, little is known about the clinical role of *COL1A1* in lung cancer.

For decades, despite advances in targeted therapies and immunotherapies, platinum-based chemotherapy has remained the standard first-line treatment for most advanced lung cancer 11. Intrinsic or acquired resistance to chemotherapy is the major cause of therapeutic failure 12. There is currently no available method to identify intrinsically or acquired chemoresistance in patients at baseline. Carcinoembryonic antigen (CEA) is a serum glycoprotein. However, it is widely used as a validated biomarker for disease monitoring and assessing the response and relapse in lung cancer; the results often lack specificity and sensitivity 13, 14. A previous study found that stellate cell-secreted *COL1A1* can promote invasion and migration of pancreatic cancer cells, which is also associated with chemoresistance 15.

In the present study, we found that the *COL1A1* level was significantly elevated in lung cancer tissues and serum samples, and it was closely associated with poor PFS and chemoresistance of lung cancer. These findings suggest that *COL1A1* might be a candidate diagnostic, prognostic, and chemoresistance biomarker for lung cancer patients.

## Materials and methods

### Materials

Anti-Collagen I antibody (ab34710) was purchased from Abcam PLC (Cambridge, UK). Human *COL1A1* enzyme-linked immunosorbent assay (ELISA) kit was purchased from Beijing Dogesce Biological Technology, Ltd. (Beijing, People's Republic of China).

### Methods

#### Analysis of Oncomine data

To determine the expression pattern of *COL1A1* in lung cancer, we used the datasets in Oncomine (https://www.oncomine.org), a cancer microarray database, and an online data-mining platform, aimed at promoting discovery from genome-wide expression analyses 16. The data cohorts of Weiss et al. deposited in Oncomine.com, which includes 155 lung squamous cell carcinomas (LSCC), 77 LUAD, and 59 normal lung tissues, were analyzed for gene copy number 17. The study of Rohrbeck et al., which included 16 LUAD, 9 small cell lung cancer (SCLC), and 5 normal lung tissues, analyzed the mRNA level 18. The data were displayed using a Box chart.

#### Human specimens

**Figure 1:**
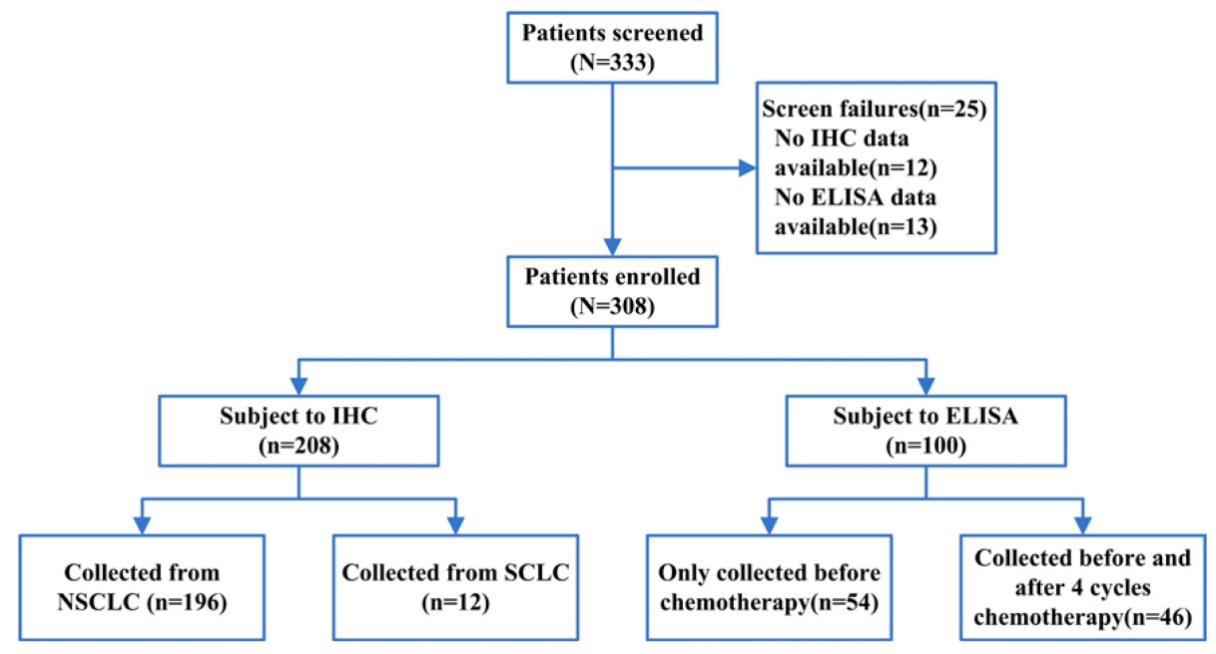
The flowchart of enrolled patients.

Gene expression analysis was successful in 308 biopsy specimens with full clinical data out of a total of 333 trial participants (**Figure 1**). A total of 208 formalin-fixed paraffin-embedded human lung cancer specimens were obtained from the Harbin Medical University Cancer Hospital. Human solid lung cancer specimens were collected from January 2018 to July 2019. All patients had histologically confirmed lung cancer with information on the histologic type and tumor stage (AJCC, The American Joint Committee on Cancer). This study, involving human lung tumor specimens, was approved by the Institutional Review Board of the Harbin Medical University Cancer Hospital.

Blood samples were obtained from 100 patients with advanced lung cancer from January 2018 to July 2019. Serum samples of 54 patients were collected before chemotherapy, and the samples of 46 patients were collected before and after four cycles of chemotherapy. In addition, 40 serum samples donated voluntarily by healthy individuals were used as a control group during the study period. Fasting peripheral venous blood (5 ml) was collected from all participants in the morning.

#### Immunohistochemistry (IHC) and hematoxylin and eosin (H&E) staining

Paraffin sections were then stained with the avidin-biotin complex IHC stain following a standard staining protocol to detect the expression of *COL1A1*. After endogenous peroxidase was quenched with 3% hydrogen peroxide and blocked for 10 min, sections were incubated overnight with the primary antibody at 4°C. Rabbit primary monoclonal antibodies directed against *COL1A1* (1:100 dilution; Abcam) were used in the experiment. Biotinylated immunoglobulin and peroxidase-conjugated streptavidin were then added. Finally, 3, 3'-diaminobenzidine was added for color development, and hematoxylin was used for counterstaining. The staining intensity and extent scored protein expression levels. Tissues with no staining were rated as 0, those with faint staining or moderate to strong staining in 10% of cells were rated as 1, those with moderate staining or strong staining in 10%-50% of cells were rated as 2, and those with strong staining in > 50% of cells were rated as 3. Cases that registered levels 0 and 1 were considered negative, and those that registered levels 2 and 3 were defined as positive 19. H&E staining was used to indicate the pathology.

#### ELISA

*COL1A1* concentration was determined using an ELISA method. The assays were performed according to the manufacturer's protocols. Furthermore, 100 μl of HRP-conjugate reagent was added to the serum samples from cancer patients and healthy controls, covered with an adhesive strip, and incubated for 60 min at 37°C. Each well was aspirated and washed, and it was washed by filling with Wash Solution (400 μl) using a squirt bottle, manifold dispenser, or auto washer. Samples of 50 μl were added to each well. They were gently mixed and incubated for 15 min at 37°C, and they were protected from light. Finally, 50 μl Stop Solution was added to each well. All of the samples were examined three times, and the median values were used for statistical analysis.

#### Assessment and statistical analysis

All of the patients had a follow-up ≥ 12 months. PFS was defined as the interval from the initial chemotherapy to disease progression or death, without any evidence of progression. Serum samples were divided into the chemosensitive (partial or complete response) and chemoresistant (progressive disease) groups based on the Response Evaluation Criteria in Solid Tumors (RECIST Edition 1.1).

All of the data were analyzed with Statistical Package for the Social Sciences version 24 software (SPSS Inc., Chicago, IL, USA). The intragroup expression difference detected by the IHC assay was analyzed by the rank-sum test. We analyzed the association between *COL1A1* and clinicopathologic parameters using the independent *t*-test, Mann-Whitney test, or Kruskal-Wallis test as appropriate. The paired *t*-test was used for ELISA group analysis. Receiver operator characteristic (ROC) curves were created to determine the predictive value of *COL1A1* and CEA to distinguish between lung cancer patients and controls. Survival curves for PFS were estimated using the Kaplan-Meier method, and the differences were evaluated with the log-rank test. Univariate and multivariate analyses of the PFS were performed using Cox-regression analysis. Continuous variables were summarized using mean and standard deviation. Statistical significance was set at* P* < 0.05.

## Results

### *COL1A1* gene amplification and elevation at mRNA levels in lung cancer

**Figure 2:**
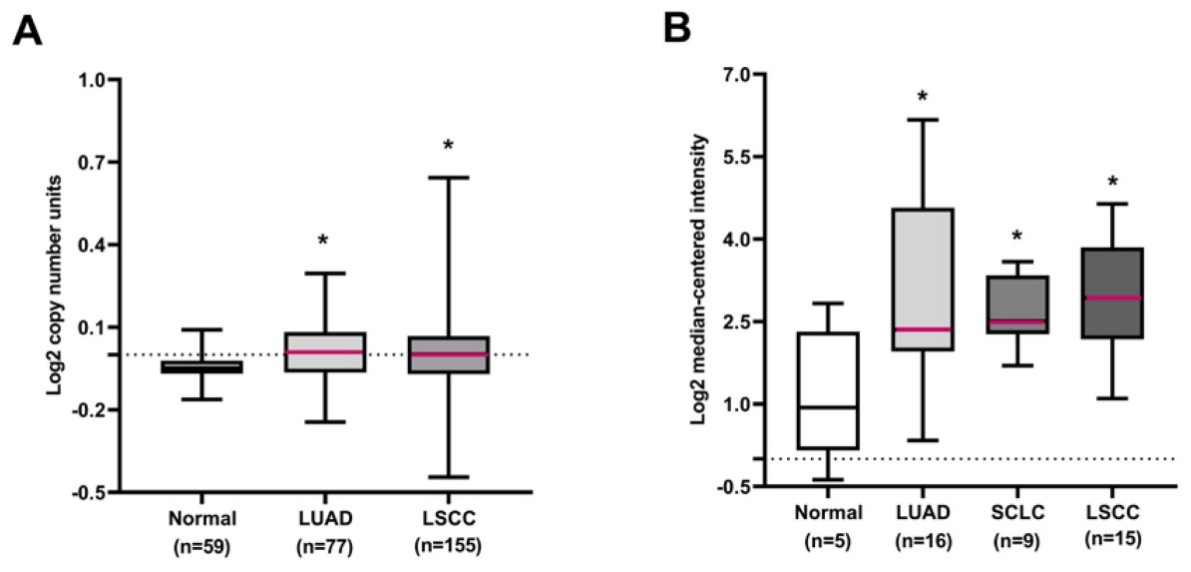
***COL1A1* DNA or mRNA expression in human lung cancers using the Oncomine database. (A)** The *COL1A1* gene copy number in normal lung tissues and different histological subtypes of lung cancer. **(B)** *COL1A1* mRNA expression in normal lung tissues and different histological subtypes of lung cancer. *P < 0.05 compared with the normal group.

To assess the clinical relevance of *COL1A1* in human primary lung cancers, the expression profile of *COL1A1* was characterized by bioinformatics analysis of public datasets from Oncomine. The results showed that the *COL1A1* gene copy number increased in lung cancer tissues compared to normal lung tissues in the Weiss lung dataset (**Figure 2A**). We also observed upregulation of the *COL1A1* mRNA level in lung cancer tissues compared with normal lung tissues in the Rohrbeck Lung dataset (**Figure 2B**). Additionally, Merry et al. identified that a characteristic amplification of sequences from chromosome 17q, demarcated by the *COL1A1* gene, was associated with elevated expression of the *COL1A1* in dermatofibrosarcoma protuberans (DFSP) 20. These observations suggested that the *COL1A1* protein may be highly expressed in lung cancer tissues.

### *COL1A1* expression in lung cancer tissues by IHC

**Figure 3:**
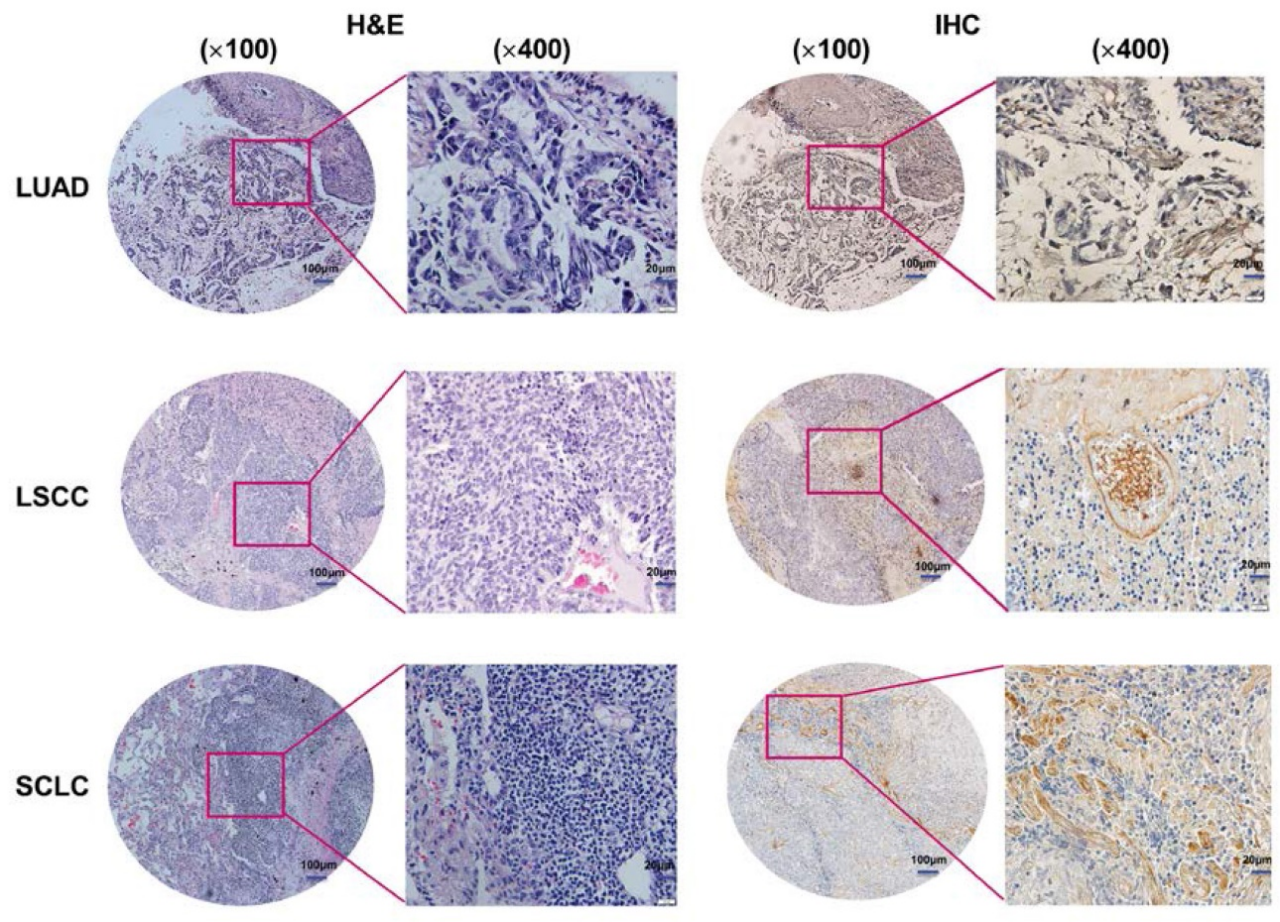
***COL1A1* expression in lung cancer tissues.** Representative H&E and IHC staining of *COL1A1* in LUAD, LSCC, and SCLC patients (magnification×100, ×400). Immunoreactivity was observed in the malignant cell-extracellular matrix.

To examine the correlation between *COL1A1* expression and clinicopathologic factors in lung cancer, we evaluated its expression in lung cancer tissues by IHC. The results showed that 110 of the 208 primary lesions exhibited positive staining for *COL1A1*, but there was no staining of the adjacent normal tissues (**Figure 3**). Statistical analysis revealed that higher *COL1A1* expression were significantly correlated with the peripheral type tumor (*P* = 0.001), larger diameter of the tumor (*P* = 0.009), occurrence of lymph node metastases (*P* = 0.009) and distant metastases (*P* < 0.001), higher TNM stage (III, IV) (*P* = 0.006), and smoking (*P* < 0.001) (**Table 1**). However, no statistically significant correlations were identified between the expression of *COL1A1* and other clinicopathologic parameters, including local invasion and pathologic type.

### Elevated serum levels of *COL1A1* in lung cancer patients

**Figure 4:**
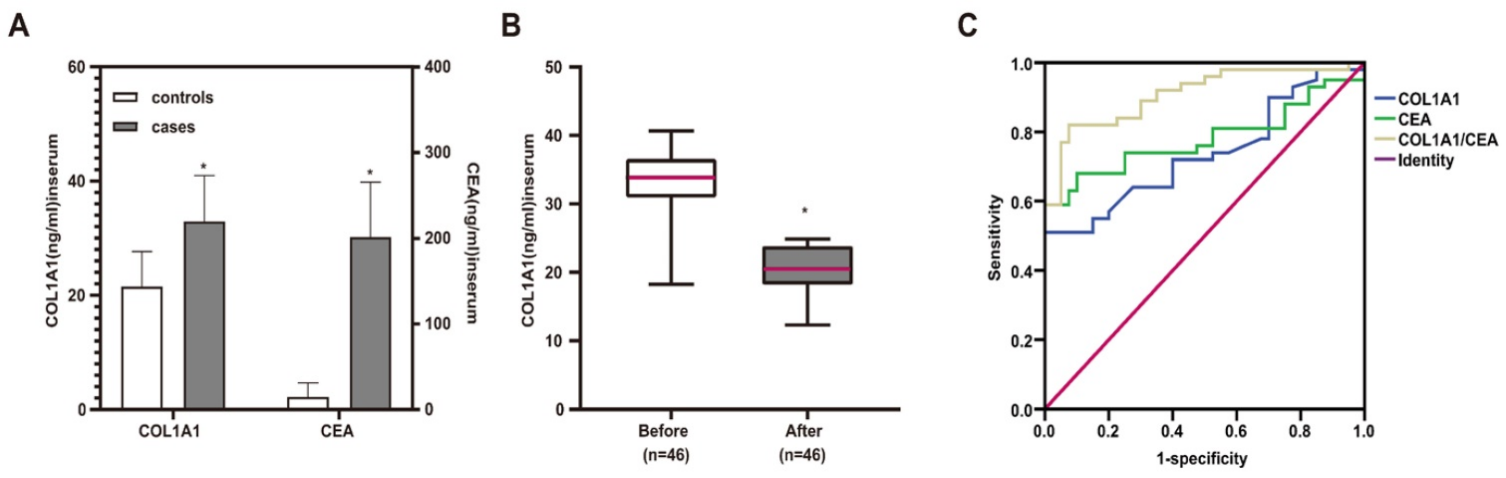
** Elevated* COL1A1* levels in lung cancer serum samples. (A)** Comparison of serum *COL1A1* and CEA levels between lung cancer patients and healthy individuals. *P < 0.05 healthy individuals vs. lung cancer patients (independent sample *t-*test)** (B)** The serum levels of *COL1A1* before and after four cycles of chemotherapy in lung cancer patients. *P < 0.05 before chemotherapy vs. after four cycles of chemotherapy (paired *t-*test) **(C)** ROC curves for the serum *COL1A1*, CEA, and the combination of *COL1A1* and CEA in the diagnosis of lung cancer.

To investigate the potential of *COL1A1* as a serological marker for lung cancer, we analyzed the level of *COL1A1* in serum from lung cancer patients (n = 100) and healthy individuals (n = 40) by ELISA. The result showed that the serum levels of *COL1A1* in lung cancer patients were significantly higher than those in the healthy group (32.90 ± 8.08 ng/ml vs. 21.51 ± 6.16 ng/ml, *P* < 0.001) (**Figure 4A**). In addition, the serum levels of *COL1A1* in lung cancer patients before chemotherapy were significantly higher than in patients after four cycles of chemotherapy (32.67 ± 5.84 ng/ml vs. 19.98 ± 3.95 ng/ml, *P* < 0.001) (**Figure 4B**).

Then, we analyzed the association between the levels of *COL1A1* in the serum of lung cancer patients and clinicopathologic parameters. As shown in **Table 2**, the higher concentrations of *COL1A1* were significantly correlated with the peripheral type tumor (*P* < 0.001), larger diameter of the tumor (*P* = 0.028), occurrence of lymph node metastases (*P* < 0.001) and distant metastases (*P* = 0.001), higher TNM stage (III, IV) (*P* = 0.049), and smoking (*P* < 0.001).

### The diagnostic value of *COL1A1* as a serologic biomarker for lung cancer patients

To further evaluate the potential clinical utility of *COL1A1* only or combined with CEA as the diagnostic serum marker, we employed the ROC curve analysis. CEA being a common serum marker, its serum levels in patients with lung cancer (30.19 ± 9.64 ng/ml) were higher than those in healthy volunteers (2.20 ± 2.47 ng/ml, *P* < 0.001) (**Figure 4A**). As shown in **Figure 4C**, the AUC of *COL1A1* was 0.737 (95% CI: 0.656-0.817), although it did not outperform the serum CEA (AUC = 0.780, 95% CI: 0.706-0.854). The combined examination of *COL1A1* and CEA had a better discriminating potential (AUC = 0.909, 95% CI: 0.861-0.957).

### Overexpression of *COL1A1* correlates with poor prognosis in lung cancer patients

**Figure 5:**
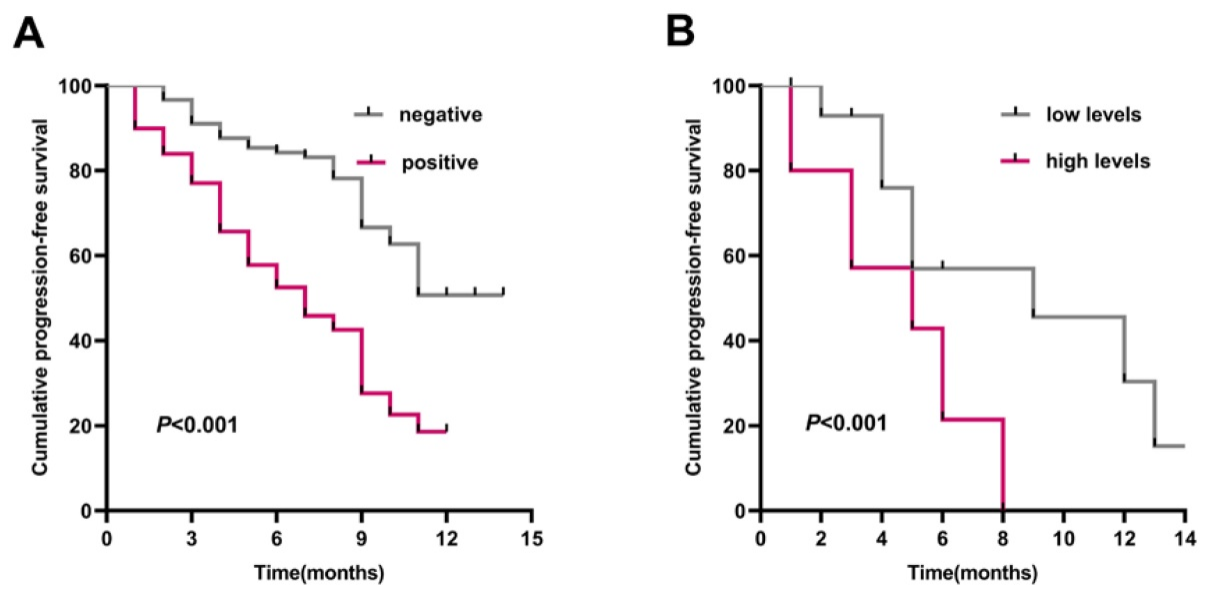
** The prognostic value of *COL1A1* expression.** Kaplan-Meier survival curves comparing lung cancer patients with high and low expression of *COL1A1*. **(A)** PFS survival curves of lung cancer patients in tissue samples.** (B)** PFS survival curves of lung cancer patients in blood samples.

To determine the correlation between *COL1A1* expression and clinical outcome, we analyzed the prognostic significance of *COL1A1* using Kaplan-Meier analysis. In the IHC group, the PFS time of lung cancer patients with *COL1A1*-positive expression was markedly shorter than in the patients with *COL1A1*-negative expression (**Figure 5A**). In the ELISA group, a cutoff value corresponding to the median of *COL1A1* was used to separate the patients into two groups: those with high serum levels (≥28.46 ng/ml) versus those with low serum levels (<28.46 ng/ml). Patients with low serum *COL1A1* levels had a median PFS of 9 months compared to patients with high serum *COL1A1* levels who had a PFS of 5 months (*P <* 0.001, log-rank test) (**Figure 5B**).

Moreover, the subsequent univariate and multivariate Cox-regression model showed that the positive of *COL1A1* (HR = 2.696; 95% CI: 1.827-3.980; *P* < 0.001), occurrence of lymph node metastases (HR = 4.586; 95% CI: 2.916-7.213; *P* < 0.001), higher local invasion at stage T3-T4 (HR = 3.483; 95% CI: 2.025-5.990; *P* < 0.001), and smoking (HR = 1.488; 95% CI: 1.039-2.132; *P* = 0.030) were statistically independent predictive factors of poorer prognosis for lung cancer patients (**Table 3**). The chemotherapy regimens for all patients with lung cancer are shown in **Table 4**.

### Discriminating potential of serum *COL1A1* to identify lung cancer patients with intrinsic chemoresistance

**Figure 6:**
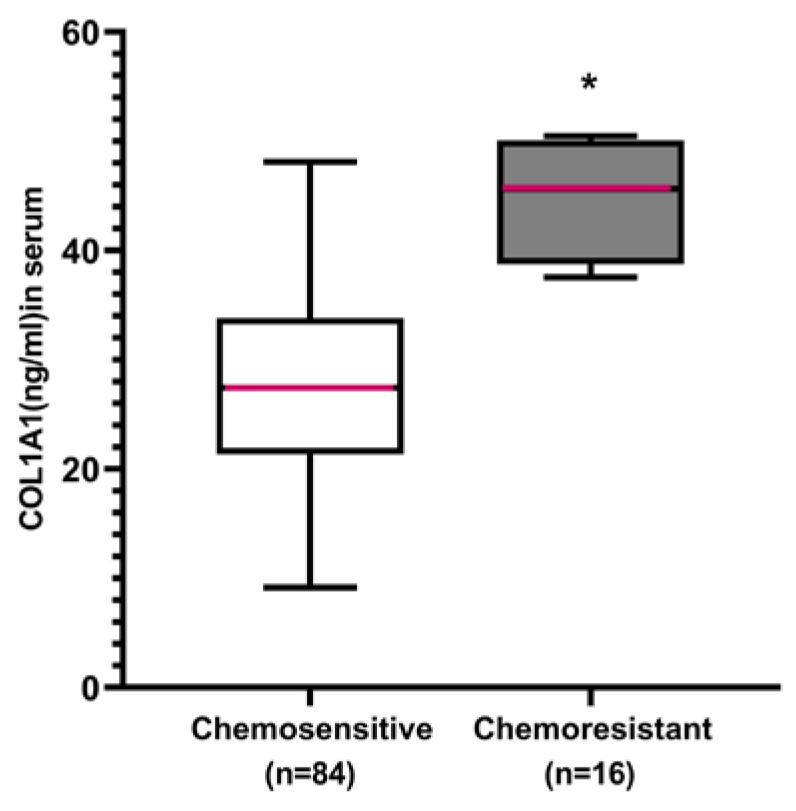
** The potential of serum *COL1A1* to identify lung cancer patients with intrinsic chemoresistance.** Box plots representing the serum level of *COL1A1* in lung cancer patients with chemoresistance and chemosensitivity to first-line chemotherapy. *P < 0.05 chemosensitive group vs. chemoresistance group (independent sample *t-*test). All experiments were repeated three times.

To further evaluate the clinical utility of *COL1A1* as the intrinsic chemoresistance serum marker, we analyzed the serum *COL1A1* levels in 100 lung cancer patients using ELISA. Of the 100 patients, 84 were chemosensitive and 16 were chemoresistant. Serum *COL1A1* levels were significantly lower in the chemo-sensitive patients (28.68 ± 9.03 ng/ml) compared with the chemoresistant patients (44.82 ± 5.35 ng/ml, *P* < 0.001) (**Figure 6**).

## Discussion

It is challenging to explore a specific biomarker for the diagnostic, prognosis, and intrinsic chemoresistance of lung cancer. Based on our previous bioinformatics analysis, genetic alterations of the extracellular matrix protein *COL1A1* are likely to be a frequent event in human lung cancers. Currently, in lung cancer, the clinical role of *COL1A1* is not sufficiently understood. In our experimental data, *COL1A1* was highly expressed in human lung cancer tissues and was correlated with some clinicopathologic parameters, such as the occurrence of lymph nodes and distant metastases. Our ELISA analysis revealed that the clinical significance of the serum level of *COL1A1* in lung cancer patients was consistent with lung cancer tissues. Furthermore, our statistical analysis revealed that *COL1A1* was associated with poor prognosis and chemoresistance of lung cancer. These data suggest that *COL1A1* can be used as a potential biomarker in the diagnosing, prognosis, and chemoresistance of human lung cancer.

*COL1A1* is one of the most abundant proteins in the human body. It is essential to maintain the structure of bone, skin, tendons, cornea, blood vessel walls, and other connective tissues 21. In addition, *COL1A1* promotes embryonic stem cell proliferation and contributes to increased extracellular environment stiffness in the vertebrates 22. It is widely accepted that *COL1A1* is produced by stromal fibroblasts 23. *COL1A1* is associated with response to hypoxia and oxidative stress, which may help the cells adapt to oxidative stress and hypoxia, thus promoting tumor cell growth and metastasis 24. Currently, in lung cancer cells, the role of *COL1A1* is not sufficiently understood. *COL1A1* is implicated in the induction of epithelial-mesenchymal transition (EMT) through the transforming growth factor (TGF)-β-dependent pathway in non-SCLC cell lines 25, 26. Therefore, blocking TGF-β signaling may be an effective treatment to prevent cancer progression in patients with non-small-cell lung cancer (NSCLC). Our data showed that *COL1A1* was highly expressed in lung cancer specimens (52.8%), which is in line with the previous reports on gastric cancer 27.

Moreover, *COL1A1* was closely associated with the overall survival of cancer patients. Boguslawska et al. found that TGF-β1 induced the expression of *COL1A1* in a renal cell carcinoma-derived cell line, and disturbed expression of *COL1A1* occurred early during renal cell carcinoma carcinogenesis and correlated with poor survival 28. Likewise, *COL1A1* has also been reported to be one component of hepatic fibrosis, and *COL1A1* gene methylation might be correlated with the poor clinical outcomes of hepatocellular carcinoma 29. In our study, higher *COL1A1* levels were significantly associated with shorter PFS, and overexpression of *COL1A1* may predict a poor prognosis in advanced lung cancer patients.

However, because of chemoresistance, lung cancer has a low five-year survival rate and a high fatality rate, leading to chemotherapy failure 30. It is critical to identify the patients who would benefit from chemotherapy. A recent study revealed that activated fibroblasts play a prominent role in lung carcinogenesis, and cancer-associated fibroblasts are responsible for deposition of extracellular matrix 31. Consequently, proteins related to the extracellular matrix are released into the blood and could be considered potential circulating biomarkers 32. Cisplatin is a cell-cycle nonspecific agent widely used in the first-line chemotherapy of lung cancer patients 33. *MiR-29b-3p* can reverse cisplatin resistance by inhibiting the expression of the *COL1A1* gene in NSCLC cell lines 34. In our study, *COL1A1* could be used as a predictive biomarker for chemoresistant patients with advanced lung cancer in first-line chemotherapy.

In conclusion, *COL1A1* can be used as a novel diagnostic, prognostic, and chemoresistance biomarker for lung cancer. These findings provide novel directions for studying the precise molecular mechanism of the *COL1A1* gene and new strategies to develop potential *COL1A1*-associated anti-lung cancer drugs.

## Figures and Tables

**Table 1:** Correlation between the *COL1A1* expression and clinicopathologic parameters in lung cancer tissues.

Cases (n)		COL1A1 average rank	Statistical test	*P*-value
Location				
Central type	108	93.15		
Peripheral type	100	116.76	Mann-Whitney test	0.001
Tumor diameter				
d≤3cm	71	90.41		
3cm<d≤7cm	113	109.42		
d>7cm	24	123.00	Kruskal-Wallis test	0.009
Lymph node metastasis				
No	66	90.70		
Yes	142	110.92	Independent *t*-test	0.009
Local invasion				
T1-T2	184	102.09		
T3-T4	24	123.00	Mann-Whitney test	0.062
Distant metastasis				
No	82	70.37		
Yes	126	126.71	Independent *t*-test	<0.001
Pathologic type				
LSCC	106	102.89		
LUAD	90	108.56		
SCLC	12	88.33	Kruskal-Wallis test	0.399
Stage				
Ⅰ-Ⅱ	61	89.33		
Ⅲ-IV	147	110.80	Mann-Whitney test	0.006
Smoking				
No	110	80.93		
Yes	98	130.96	Independent *t*-test	<0.001

**Table 2:** Correlation between the *COL1A1* level and clinicopathologic parameters in the serum of lung cancer patients.

Cases (n)		COL1A1(ng/ml)	Statistical test	*P*-value
Location				
Central type	39	31.27±7.18		
Peripheral type	61	33.78±8.19	Mann-Whitney test	<0.001
Tumor diameter				
d≤3cm	45	30.55±6.95		
3cm<d≤7cm	43	34.49±10.53		
d>7cm	12	35.40±5.76	Kruskal-Wallis test	0.028
Lymph node metastasis				
No	30	29.31±5.82		
Yes	70	34.51±7.65	Independent *t*-test	<0.001
Local invasion				
T1-T2	82	33.89±6.67		
T3-T4	18	35.58±8.62	Mann-Whitney test	0.424
Distant metastasis				
No	24	30.63±8.04		
Yes	76	33.16±7.55	Mann-Whitney test	0.001
Pathologic type				
LSCC	15	32.90±5.52		
LUAD	64	34.27±7.41		
SCLC	21	31.93±8.41	Kruskal-Wallis test	0.790
Stage				
Ⅰ-Ⅱ	12	28.46±6.63		
Ⅲ-IV	88	34.07±4.61	Independent *t*-test	0.049
Smoking				
No	49	30.94±7.35		
Yes	51	35.28±8.18	Independent *t*-test	<0.001

**Table 3:** Univariate and multivariate analyses of Cox proportional hazard regression model for PFS in lung cancer patients.

Pathologic parameters	PFS		
	HR	95% CI	*P*-value
**Univariate analysis**			
COL1A1			
Positive vs Negative	2.732	1.863-4.006	<0.001
Location			
Central type vs Peripheral type	0.965	0.679-1.371	0.841
Tumor diameter			
d≤3cm vs 3cm<d≤7cm vs d>7cm	1.849	1.380-2.476	<0.001
Lymph node metastasis			
Yes vs No	4.481	2.823-7.111	<0.001
Local invasion			
T1-T2 vs T3-T4	2.470	1.476-4.136	0.001
Distant metastasis			
Yes vs No	1.828	1.259-2.655	0.002
Pathologic type			
LSCC vs LUAD vs SCLC	0.997	0.754-1.317	0.981
Stage			
Ⅰ-Ⅱ vs Ⅲ-IV	4.340	2.691-6.998	<0.001
Smoking			
Yes vs No	1.434	1.007-2.042	0.046
**Multivariate analysis**			
COL1A1			
Positive vs Negative	2.696	1.827-3.980	<0.001
Tumor diameter			
d≤3cm vs 3cm<d≤7cm vs d>7cm	1.400	0.913-2.145	0.205
Lymph node metastasis			
Yes vs No	4.586	2.916-7.213	<0.001
Local invasion			
T1-T2 vs T3-T4	3.483	2.025-5.990	<0.001
Distant metastasis			
Yes vs No	1.526	0.977-2.384	0.099
Stage			
Ⅰ-Ⅱ vs Ⅲ-IV	0.536	0.211-1.361	0.293
Smoking			
Yes vs No	1.488	1.039-2.132	0.030

**Table 4:** Chemotherapy regimens for lung cancer patients.

Type of lung cancer	Chemotherapy regimens
NSCLC	Pemetrexed + cisplatin
	Gemcitabine + cisplatin
	Paclitaxel + cisplatin
	Vinorelbine + cisplatin
	Docetaxel + cisplatin
	Pemetrexed + carboplatin
	Gemcitabine + carboplatin
	Paclitaxel + carboplatin
	Gemcitabine
	Docetaxel
SCLC	Etoposide + cisplatin
	Etoposide + carboplatin
	Irinotecan + cisplatin
	Paclitaxel
	Docetaxel
	Gemctabine
